# Clinical Characteristics and Management of Children and Adolescents Hospitalized With Pyomyositis

**DOI:** 10.1097/INF.0000000000004382

**Published:** 2024-05-16

**Authors:** Sebastian Weber, Chloé Schlaeppi, Florence Barbey, Michael Buettcher, Beate Deubzer, Andrea Duppenthaler, Manon Jaboyedoff, Christian Kahlert, Lisa Kottanattu, Christa Relly, Noemie Wagner, Petra Zimmermann, Ulrich Heininger

**Affiliations:** From the * Faculty of Medicine, University of Basel; † Department of Paediatric Infectious Diseases and Vaccinology, University Children’s Hospital Basel (UKBB), Basel; ‡ Division of Infectious Diseases, Children`s Hospital & Department of Paediatrics, Cantonal Hospital Aarau, Aarau; § Paediatric Infectious Diseases, Lucerne Children’s Hospital; ¶ Faculty of Health Science and Medicine, University Lucerne, Lucerne; ∥ Paediatric Pharmacology and Pharmacometrics Research Centre, University Children’s Hospital Basel (UKBB), Basel; ** Paediatric Infectious Diseases, Children’s Hospital, Cantonal Hospital of Grisons, Chur; †† Paediatric Infectious Diseases Unit, Department of Paediatrics, Inselspital Bern, University Hospital, University of Bern, Bern; ‡‡ Paediatric Infectious Diseases and Vaccinology Unit, Service of Paediatrics, Department Mother-Woman-Child, Lausanne University Hospital and University of Lausanne, Lausanne; §§ Department of Infectious Diseases and Hospital Epidemiology, Children’s Hospital of Eastern Switzerland, St. Gallen; ¶¶ Institute of Paediatrics of Southern Switzerland, Ospedale Regionale di Bellinzona e Valli, Bellinzona; ∥∥ Division of Infectious Diseases and Hospital Epidemiology, University Children’s Hospital Zurich, Zurich; *** Paediatric Infectious Diseases Unit, Children’s Hospital, Geneva University Hospitals and Faculty of Medicine, Geneva; ††† Department of Paediatrics, Fribourg Hospital Fribourg; ‡‡‡ Department of Community Health, Faculty of Science and Medicine, University of Fribourg, Fribourg, Switzerland.

**Keywords:** pyomyositis, bacterial myositis, osteomyelitis, septic arthritis

## Abstract

**Background:**

Pyomyositis, a bacterial muscle infection, is an important differential diagnosis in children and adolescents with musculoskeletal pain. In contrast to tropical regions, it is rarely recognized in temperate countries, but incidence is increasing and major studies are missing.

**Methods:**

This retrospective multicenter study included patients <18 years of age hospitalized with pyomyositis in 11 Swiss children’s hospitals between January 2010 and December 2022. Cases were identified by ICD-10 code (Myositis; M60–M60.9), and data was extracted from electronic hospital records.

**Results:**

Of 331 patients identified, 102 fulfilled the case definition. Patient age at presentation ranged from 2 weeks to 17 years (median 8 years). The majority had no underlying illness and all presented with fever and localized pain. At the respective site of pyomyositis, 100 (98%) had impaired movement and 39 (38%) presented with local swelling. Pelvic (57%) and leg (28%) muscles were mostly affected. Blood or tissue cultures were obtained in 94 (92%) and 59 (57%) patients, respectively. Of those, 55 (58%) blood and 52 (88%) tissue cultures were positive, mainly for *Staphylococcus aureus* (35 and 19, respectively) and *Streptococcus pyogene*s (12 and 15, respectively). All patients received antibiotic treatment during hospitalization for a median of 10 days (interquartile range: 7–17), followed by outpatient treatment for a further median of 16 days (interquartile range: 11–22) in 95 (93%) patients. Fifty-nine (57%) patients required surgery.

**Conclusions:**

Pyomyositis is a challenging diagnosis that requires a high level of awareness. Blood and/or tissue cultures revealed *S. aureus* and *S. pyogenes* as the predominant causative agents.

Pyomyositis is an acute bacterial infection of the skeletal muscle with local abscess formation.^1^ Initially described as endemic to tropical regions and thus referred to as “tropical pyomyositis”^2^ it is now increasingly reported globally.^2–10^ Pyomyositis is thought to result from a combination of muscle injuries and bacteremia.^6^ Skeletal muscle gets susceptible to hematogenous bacterial invasion once damaged.^11^ Pyomyositis is a diagnostic challenge due to its nonspecific clinical presentation^5,12^ and rare occurrence. Prompt diagnosis and treatment is advisable to decrease the risk of sequelae.^6^

Pyomyositis occurs in all age groups but the majority of cases have been reported in children and adolescents.^13^ In the pediatric population, mainly healthy children are affected^14^ whereas in adults it is often reported in immunocompromised individuals,^15^ including people living with HIV.^1^ Due to its rarity, large reviews include mixed pediatric and adult populations.^1,15^ For the pediatric population, the available literature consists mainly of case reports or series^3,5,7–9,14,16–22^ and one systematic review of case series.^4^

The aim of this retrospective study was to describe the clinical presentation, diagnosis, management, and short-term outcome of pyomyositis in children and adolescents.

## METHODS

### Patient Population

The study was conducted in 11 pediatric hospitals in Switzerland. Members of the Pediatric Infectious Disease Group of Switzerland (https://pigs.ch/) were invited and 11 of 13 centers participated. These included all 5 university children’s hospitals (Basel, Bern, Geneva, Lausanne and Zurich) and 6 cantonal tertiary children’s hospitals (Aarau, Bellinzona, Chur, Fribourg, Lucerne and St. Gallen).

### Study Design

We performed a retrospective chart review of patients under 18 years of age hospitalized with pyomyositis at a collaborating center between January 1, 2010 and June 30, 2022. For the study site in Basel, the study period was extended until January 31, 2023. Due to the retrospective, descriptive study design, no patient consent was obtained. The study was approved by the lead ethics committee of Northwest-Central Switzerland (EKNZ Number 2022–01341) for Basel, Lucerne, Aarau as well as all local ethics committees of the other participating hospitals (Bern, Geneva, St. Gallen, Lausanne and Zurich) on June 21, 2022.

Patients were identified through an electronic hospital database search for the diagnosis of “myositis” (ICD-10 codes M60.0–60.9) and included if no refusal to use their data for research purposes was documented.

### Data Collection

Data were extracted from the electronic medical records at the respective hospitals by onsite visits from a study group member (SW) and managed using REDCap electronic data capture tools hosted at the University of Basel Children’s Hospital.^23,24^

We obtained demographic information, predisposing factors such as underlying illness, immunosuppression, trauma, viral infection, travel history and clinical signs or symptoms on admission. Further, information about dates of hospital admission and discharge (and readmission to the same hospital, if applicable), intensive care unit stay, pyomyositis-related complications, laboratory and radiographic information and data on antibiotic and surgical treatment were collected.

### Definitions

We created a case definition to discriminate pyomyositis from bacterial myositis and pyomyositis associated with osteomyelitis (OM) or septic arthritis (SA), classified as follows:

1. Pyomyositis: compatible clinical presentation with radiographic detection of abscess formation, with or without bacterial pathogen detected by blood or tissue culture (s).
2. Bacterial myositis: compatible clinical presentation with radiographic signs of myositis but without abscess, with bacterial pathogen detected by blood or tissue culture (s) and without an alternative diagnosis.
3. Pyomyositis associated with OM or SA: criteria for pyomyositis fulfilled in the presence of OM or SA.

Fever was defined as body temperature ≥38.0 °C. Tissue cultures (TC) were defined as cultures from punctures, intraoperative biopsies and/or, in the case of *Kingella kingae*, positive polymerase chain reaction (PCR) from throat swabs.^25^

### Statistical Analysis

Baseline data were summarized using descriptive statistics stratified by classification of pyomyositis. Analysis was conducted in R (https://www.R-project.org/) and figures were produced using the package ggplot2 (https://ggplot2.tidyverse.org). Due to limited patient numbers, we did not perform formal statistical modeling on subgroups.

## RESULTS

### Patient Characteristics

**FIGURE 1.**
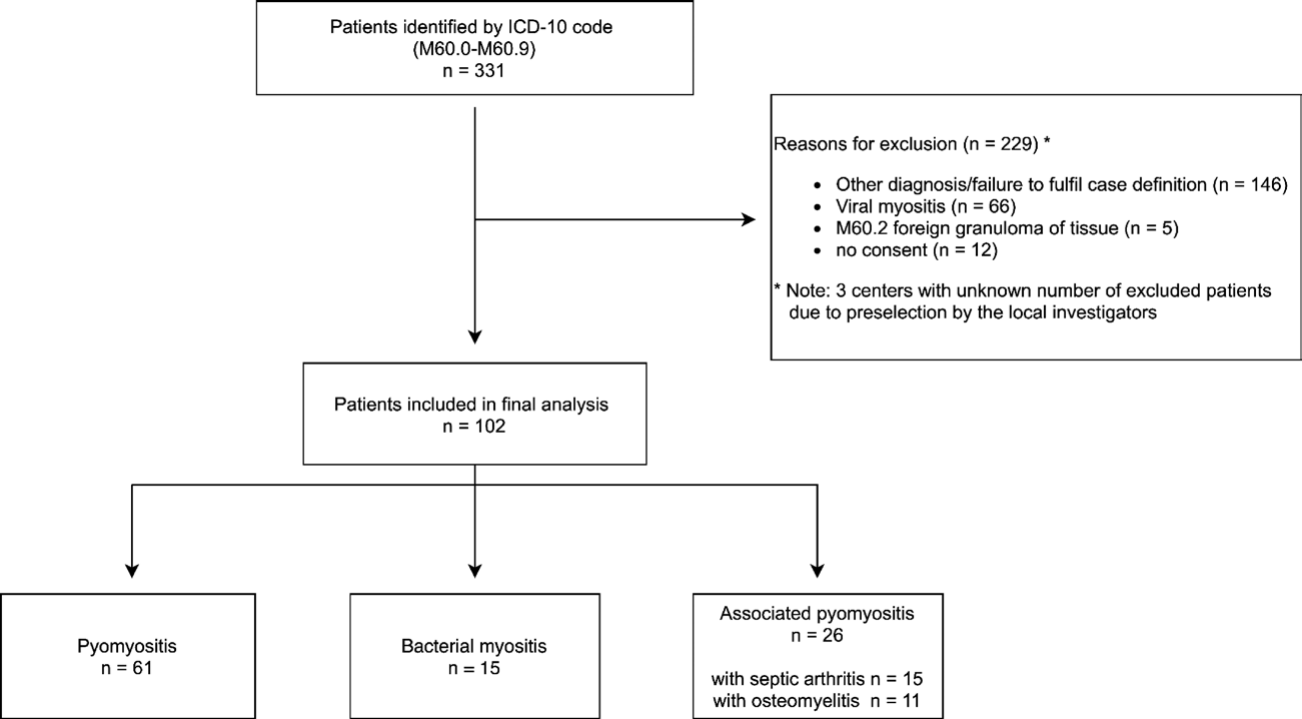
Flow chart of the study population.

A total of 331 patient records were identified, of which 102 patients (31%) from 10 hospitals were included in the final analysis (Fig. 1). Search for cases in one cantonal hospital remained without result. Of the 229 excluded patients most had an alternative diagnosis (eg, viral myositis) and failed to fulfill the pyomyositis and bacterial myositis case definitions.

Patient age ranged from 2 weeks to 17 years and included 3 neonates (2 with associated, 1 with bacterial myositis), the median age was 8 years [interquartile range (IQR): 3.9–12.6] (Table 1). Both sexes were affected equally. The majority (87%) of patients did not have an underlying disease or immunosuppressive treatment. Within 4 weeks prior to hospitalization, one-third of the children had a reported trauma (usually at the site of consequent pyomyositis). Fifty-seven (56%) had a preceding viral infection, including 4 with varicella. The median duration of symptoms prior to admission was 3 days (IQR: 2–6).

**TABLE 1.** Patient Characteristics, Clinical Presentation and Course of Disease

	Pyomyositis (n = 61)	Bacterial Myositis (n = 15)	Associated Pyomyositis (n = 26)	Total (n = 102)
General characteristics				
Male, N (%)	32 (52%)	12 (80%)	13 (50%)	57 (56%)
Median age (years) [IQR]	6.9 [3.4–10.7]	13.1 [6–13.6]	8.9 [4.9–12.2]	7.9 [3.9–12.6]
Median duration of symptoms before presentation (days) [IQR]	3 [2–6]	3 [2–4]	3 [2–6]	3 [2–6]
Predisposing factors				
Underlying illness*, N (%) -Malignancy, N (%)	8 (13%) 4 (7%)	3 (20%) 1 (6%)	2 (8%) 1 (4%)	13 (13%) 6 (6%)
Prior trauma <4 weeks†, N (%)	18 (30%)	4 (27%)	10 (39%)	32 (31%)
Prior viral infection <4 weeks, N (%)	36 (59%)	8 (53%)	13 (50%)	57 (56%)
Prior travel outside Europe <6 months, N (%)	5 (8%)	1 (7%)	0	6 (6%)
Patients with any predisposing factor, N (%)	52 (85%)	12 (80%)	20 (77%)	84 (82%)
Presentation on admission				
Localized pain, N (%)	61 (100%)	15 (100%)	26 (100%)	102 (100%)
Fever, N (%)	60 (98%)	15 (100%)	26 (100%)	101 (99%)
Localized swelling, N (%)	24 (39%)	4 (25%)	11 (42%)	39 (38%)
Impaired movement, N (%)	61 (100%)	14 (93%)	25 (96%)	100 (98%)
Walking impaired, N (%)	24 (39%)	6 (40%)	6 (23%)	36 (35%)
Refuses to walk, N (%)	27 (45%)	4 (27%)	15 (58%)	46 (45%)
Other movement restriction‡, N (%)	10 (16%)	4 (27%)	4 (15%)	18 (18%)
Involved muscle group§				
Pelvic, N (%)	34 (56%)	9 (60%)	15 (58%)	58 (57%)
Lower extremity, N (%)	17 (28%)	3 (20%)	9 (35%)	28 (28%)
Upper extremity, N (%)	9 (15%)	3 (20%)	3 (12%)	15 (15%)
Back, N (%)	4 (7%)	0	2 (8%)	6 (6%)
Others¶, N (%)	3 (5%)	0	2 (8%)	5 (5%)
Course of disease				
Median duration of hospitalization, day [IQR]	9 [6–14]	8 [6.5–10]	11 [8–19.5]	9 [7–15]
Any surgical intervention‖, N (%) Image-guided abscess puncture, N (%) Abscess incision, N (%) Joint puncture, N (%) Other intervention**, N (%)	40 (66%) 24 (40%) 14 (23%) 6 (10%) 7 (12%)	2 (13%) 0 0 1 (7%) 1 (7%)	17 (63%) 6 (23%) 7 (27%) 6 (23%) 2 (8%)	59 (58%) 30 (29%) 21 (20%) 13 (13%) 10 (10%)
ICU admissions, N (%)	9 (15%)	2 (13%)	6 (23%)	17 (17%)
Complications††, N (%)	10 (16%)	2 (13%)	0	12 (12%)
Osteomyelitis‡‡, N (%)	3 (5%)	2 (13%)	0	5 (5%)
Recurrent abscess, N (%)	2 (3%)	0	0	2 (2%)
Sepsis, N (%)	2 (3%)	0	0	2 (2%)
Sacroiliitis‡‡, N (%)	1 (2%)	0	0	1 (1%)
Necrotizing fasciitis, N (%)	1 (2%)	0	0	1 (1%)
Wound healing disorder, N (%)	1 (2%)	0	0	1 (1%)
Readmission due to pyomyositis, N (%)	6 (10%)	3 (19%)	2 (13%)	11 (11%)
Median hospitalization duration for readmissions, day [IQR]	12 [8.3–20.3]	10 [9.5–11.5]	10.5 [9–12.3]	12 [8–13.5]

* Malignancy (n = 5), M. Crohn (n = 2), malignancy and trisomy 21 (n = 1), trisomy 21 (n = 1), respiratory (n = 1), hematological (n = 1), neurological (n = 1), Ehlers–Danlos syndrome (n = 1).

† Contusions (n = 13), sport injuries or rigorous exercise (n = 9), preceding medical interventions (n = 6: minor surgical procedures n = 3, injections n = 2, bone marrow puncture n = 1) and superficial skin injuries (n = 4).

‡ Nonwalking infants with restricted movement of the lower extremities or patients with limited movement of upper limb.

§ More than one muscle was affected in 37 patients.

¶ M. rectus abdominis (n = 2), M. obliquus externus (n = 1), M. sternocleidomastoideus (n = 1), M. pectoralis major (n = 1).

‖ More than one surgical procedure was performed on several patients.

** Laparoscopies (n = 4), biopsies (n = 3), arthrotomies (n = 2), debridement (n = 1).

†† More than one complication possible.

‡‡ Defined as a complication when not present on initial MRI imaging.

ICU indicates intensive care unit.

There was a trend for increasing cases in recent years with a median of 6 cases (IQR: 4.5–9) per year from 2010 to 2016 and 10 cases (IQR: 7.5–11) between 2017 and 2022. There was no seasonality with similar case numbers in the cold (October–March, n = 48) and warm season (April–September, n = 54). Admission diagnosis was infectious myositis (ICD-10 Code M60–M60.9) in 21 (21%) of patients and OM or SA in 27 (26%) and 23 (23%) of patients, respectively. Six (6%) patients were admitted for suspected sepsis and 5 (5%) for suspected acute abdomen.

### Clinical Presentation

Most patients (97%) presented with a triad of fever, localized pain and impaired movement (Table 1). Further, local swelling was documented in the region of the affected muscle in 39 (38%) of patients. The swelling was observed more often with pyomyositis in the lower (20/28) and upper extremities (11/15) compared to the pelvic muscles (6/58). The pelvis and proximal lower extremity were the most affected localizations. Overall, the most involved muscles were M. iliopsoas (n = 20), M. obturatorius internus (n = 16) and M. quadriceps (n = 16) and while younger children were more likely to have pyomyositis in their extremities, pelvic muscle involvement was more frequent in adolescents (see Figure, Supplemental Digital Content 1, http://links.lww.com/INF/F544). In 36% of patients pyomyositis was multifocal. They did not differ from patients with unifocal pyomyositis regarding culture positivity rate, pathogen or patient age (data not shown).

### Laboratory and Microbiologic Findings

Blood (BC) or TC were obtained in 94 (92%) and 59 (58%) patients, respectively. 59% of BC were positive, with *Staphylococcus aureus* (64% of all positive BC) and *Streptococcus pyogenes* (22%) being the most commonly isolated organisms. Of TC, 86% were positive (*S. aureus* 39%, *S. pyogenes* 27%). Corresponding diagnosis and laboratory values are shown in Table 2.

**TABLE 2.** Laboratory Values and Diagnosis Category by Pathogen

Pathogen	*Staphylococcus aureus*	*Streptococcus pyogenes*	*Kingella kingae*	*Escherichia coli*	*Streptococcus pneumoniae*	Others*	Total
Number positive (% of positives)	42 (49%)	20 (24%)	6 (7%)	4 (5%)	4 (5%)	7 (10%)	83/102 (81%)
Patient age (years), median [IQR]	11.1 [6.8–13.5]	6 [3–8.9]	1 [0.9–1.5]	15.2 [14–16.6]	6.3 [0.9–13]	6.7 [4.9–14]	7.9 [3.9–12.6]
Diagnosis category Pyomyositis (% of positives)	19 (43%)	13 (30%)	3 (7%)	3 (7%)	1 (2%)	5 (11%)	44/61 (72%)
Bacterial myositis (% of positives)	10 (66%)	3 (20%)	1 (7%)	1 (7%)	0	0	15/15 (100%)†
Associated pyomyositis (% of positives)	12 (52%)	4 (17%)	2 (9%)	0	3 (13%)	2 (9%)	23/26 (88%)
Median of maximal WBC value (×10^9^/L) [IQR]	13 [10–19]	18 [15–22]	11 [10–14]	13 [5–20]	19 [17–22]	15 [9–18]	16 [11–20]
Median of maximal CRP (mg/L) [IQR]	142 [84–185]	164 [104–241]	64 [54–74]	167 [119–209]	254 [189–319]	56 [49–70]	131 [71–191]
Median of maximal ESR value (mm/h) [IQR]	60 [35–88]	50 [25–89]	64 [46–73]	40 [30–40]	72 [63–79]	58 [49–63]	58 [35–85]

* *Staphylococcus lugdunensis* (n = 1); *Enterobacter* spp. (n = 1); Group C streptococcus (n = 1); *Candida parapsilosis* and *Enterococcus* spp. (n = 1); *Streptococcus agalactiae* (n = 1); Salmonella Group D (n = 1); polymicrobial infection (self-contaminated peripherally inserted central catheter line; n = 1).

† Positive culture was a condition for case definition of bacterial myositis.

Blood culture positivity rate increased with patient age from 28% in children ≤4 years to 87% in those ≥11 years (Table 3). The spectrum of isolated pathogens also depended on patient age with more *K. kingae* and *S. pyogenes* infections in those ≤4 years (29% and 33% of all isolates, respectively) compared to those ≥5 years (0% and 19%, respectively). Of note, 3 of the 6 *K. kingae* cases were only PCR positive in throat swabs and should conservatively be considered as presumptive cases. In contrast, *S. aureus* infections predominated in older age groups (53% in those ≥5 years versus 24% in ≤4 years).

**TABLE 3.** Pathogen Detection and Laboratory Values by Age Groups

Patient Age	0–4 Years n = 32	5–10 Years n = 37	≥11 Years n = 33	Total n = 102
Pathogen detection				
Blood culture				
N positive/N specimens (% positivity rate)	8/29 (28%)	21/35 (60%)	26/30 (87%)	55/94 (59%)
Pathogen (% of all positive cultures)	*S. aureus*: n = 4 (50%) *S. pyogenes*: n = 2 (25%) *S. pneumoniae*: n = 1 *Micrococcus luteus*: n = 1	*S. aureus*: n = 11 (52%) *S. pyogenes*: n = 9 (43%) *Salmonella Gr. D*: n = 1	*S. aureus*: n = 20 (77%) *E. coli*: n = 3 (12%) *S. pyogenes*: n = 1 Others: n = 2*	*S. aureus*: n = 35 (64%) *S. pyogenes*: n = 12 (22%) *E. coli*: n = 3 (6%) Others: n = 5 (11%)
Tissue culture				
N positive/N specimen (% positivity rate)	17/21 (81%)	18/20 (90%)	16/18 (89%)	51/59 (86%)
Pathogen (% of all positive cultures)	*S. pyogenes*: n = 6 (35%) *Kingella kingae*†: n = 6 (35%) *S. pneumoniae*: n = 2 (12%) *S. aureus*: n = 2 (12%) Others: n = 1‡ (6%)	*S. aureus*: n = 9§ (50%) *S. pyogenes*: n = 7 (39%) *Enterobacter* spp.: n = 1 (5%) Others: n = 1¶ (5%)	*S. aureus*: n = 9 (56%) *E. coli*: n = 3 (19%) *S. pneumoniae*: n = 2 (13%) *S. pyogenes*: n = 1 (6%) *S. lugdunensis*: n = 1(6%)	*S. aureus*: n = 20§ (39%) *S. pyogenes*: n = 14 (27%) *Kingella kingae*†: n = 6 (12%) *S. pneumoniae*: n = 4 (8%) Others: n = 7 (14%)
Any				
N patients with any positive (% of all patients)	21 (66%)	30 (82%)	32 (97%)	83 (81%)
Pathogen (% of all positive cultures)	*S. pyogenes*: n = 7 (33%) *Kingella kingae*: n = 6 (29%) *S. aureus*: n = 5 (24%) *S. pneumoniae*: n = 2 (10%) Others: n = 1 (5%)	*S. aureus*: n = 16 (53%) *S. pyogenes*: n = 11 (37%) Others: n = 3 (10%)	*S. aureus*: n = 21 (66%) *E. coli*: n = 4 (13%) *S. pyogenes*: n = 2 (6%) *S. pneumoniae*: n = 2 (6%) Others: n = 3 (9%)	*S. aureus*: n = 42 (51%) *S. pyogenes*: n = 20 (24%) *Kingella kingae*: n = 6 (7%) *S. pneumoniae*: n = 4 (5%) *E. coli*: n = 4 (5%) Others: n = 7 (8%)
Laboratory values				
WBC N patients with ≥1 done (% of all patients)	32 (100%)	37 (100%)	32 (97%)	101 (99%)
Baseline mean WBC on admission (×10^9^/L), median [IQR]	17.7 [12.9–23.2]	14.4 [9.8–18.6]	10.25 [7–14.4]	13.95 [8.9–19.4]
Maximal mean WBC value (×10^9^/L), median [IQR]	17.7 [13.8–23.2]	15.5 [10.6–20.1]	12.4 [9.5–18.3]	15.8 [10.7–19.9]
CRP N patients with ≥1 done (% of all patients)	32 (100%)	37 (100%)	33 (100%)	102 (100%)
Baseline mean CRP on admission (mg/L), median [IQR]	71 [51–129]	97 [52–155]	100 [44–152]	90 [50.3–149.3]
Maximal mean CRP (mg/L), median [IQR]	99.5 [65.2–147.8]	129 [78–221]	162 [84–213.1]	130.5 [71.3–190.8]
ESR N patients with ≥1 done (% of all patients)	22 (69%)	26 (70%)	24 (72%)	72 (71%)
Baseline mean ESR on admission(mm/h), median [IQR]	63.5 [47–83.75]	53.5 [35–67.8]	36 [17.75–60.75]	52.5 [27.3–68.3]
Maximal mean ESR value (mm/h), median [IQR]	67 [48–85]	52 [36–74]	48.5 [26.3–84]	57.5 [35–84.5]

* Others: Concomitant *Candida parapsilosis* and *Enterococcus* spp. (n = 1); polymicrobial infection (self-contaminated peripherally inserted central catheter line; n = 1).

† Abscess puncture plus PCR positive throat swab (n = 2), joint puncture plus PCR positive throat swab (n = 1); PCR positive throat swabs only (n = 3; see Methods section).

‡ Others: *Streptococcus agalactiae* (n = 1).

§ Concomitant *Klebsiella pneumoniae* isolation from tissue culture in one patient.

¶ Others: Group C streptococcus.

C-reactive protein, erythrocyte sedimentation rate and white blood cell count median values were higher in patients with OM and SA-associated pyomyositis compared to those with pyomyositis alone (see Figures, Supplemental Digital Content 2–4, http://links.lww.com/INF/F545). Differences regarding laboratory values were also noticed depending on the age of the patients and the isolated pathogen (Table 3).

### Diagnostic Radiologic Imaging

**FIGURE 2.**
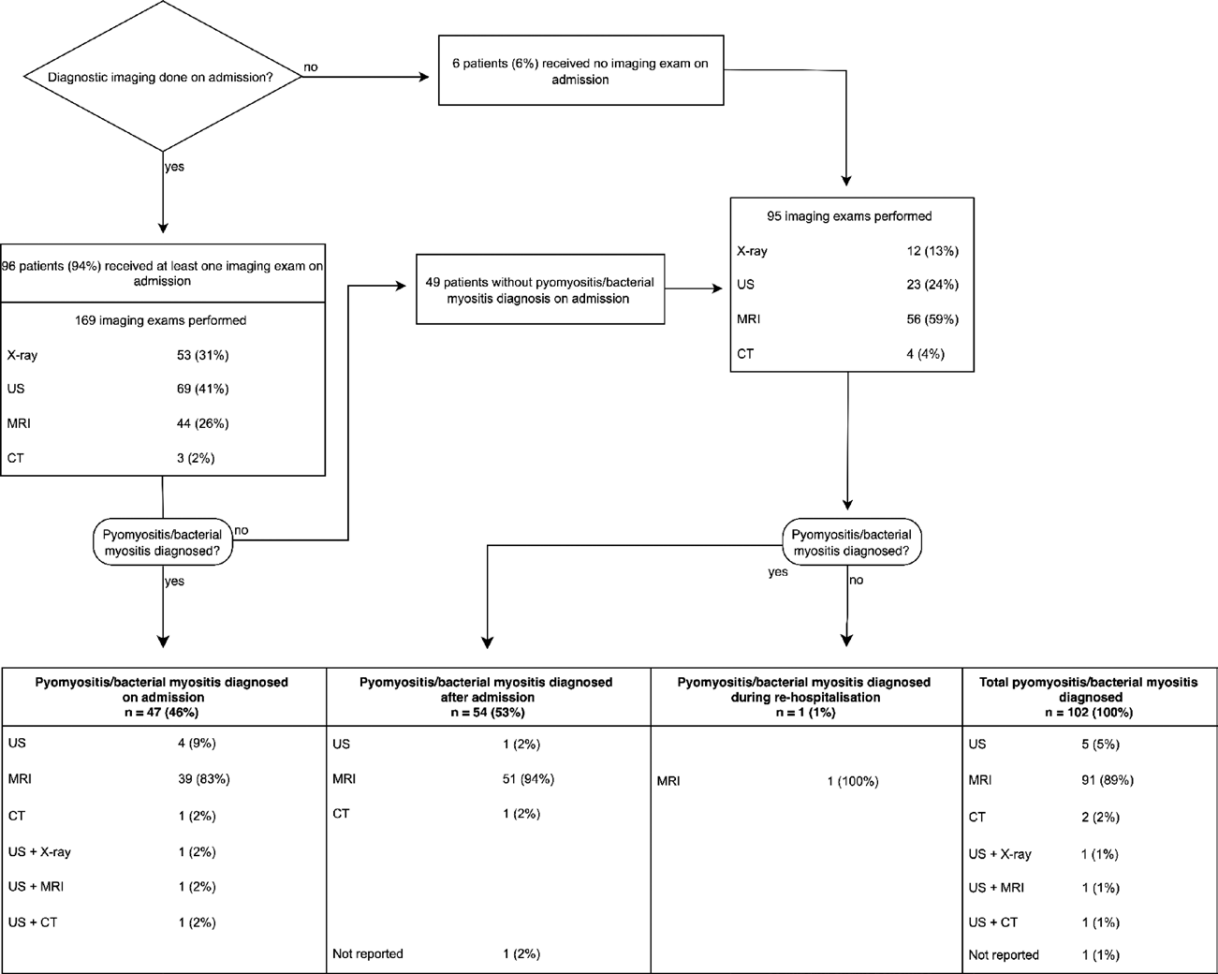
Diagnostic imaging.

Radiologic exams were performed in 96 (94%) of 102 patients on admission. In 47 (49%) of these 96 patients, the findings were compatible with bacterial myositis or pyomyositis (muscle changes or muscle abscess, respectively), with different detection rates depending on the modality (Table 4). If pyomyositis was diagnosed in the initial imaging study (n = 47) (Fig. 2), further radiological exams during hospitalization (n = 51) were mainly follow-up images (n = 41) or image-guided interventions (n = 7). If bacterial myositis or pyomyositis was not diagnosed on admission (n = 49), 95 additional images were performed during hospitalization. In 46 of these 49 patients, magnetic resonance imaging (MRI) was applied next. One case was not diagnosed during the initial admission but only on rehospitalization. In the remaining 6 patients with no imaging study on admission, a radiologic exam was performed later during their hospitalization; pyomyositis was diagnosed by MRI in 4 patients, by computed tomography (CT) in one patient and remained unproven in one patient.

**TABLE 4.** Summary of Pathological Findings on Admission in Ultrasound, Radiograph, Magnetic Resonance Imaging and Computed Tomography in 102 Patients

	US	X-ray	MRI	CT
Performed in N (%) patients	69 (68%)	53 (52%)	44 (43%)	3 (3%)
Findings: -Skin and soft tissue changes	8 (11%)	4 (8%)	5 (11%)	2 (67%)
-Muscle and tendon changes	10 (14%)	0	32 (73%)	2 (67%)
-Abscess collection	9 (13%)	1 (2%)	34 (77%)	2 (67%)
-Joint effusion and synovial swelling	6 (8%)	3 (6%)	10 (23%)	1 (33%)
-Bone and periost changes	0	2 (4%)	8 (18%)	0
-Unrelated pathology	2 (3%)	0	1 (2%)	1 (33%)
-No pathology	46 (64%)	46 (87%)	1 (2%)	0

### Antibiotic Treatment

**FIGURE 3.**
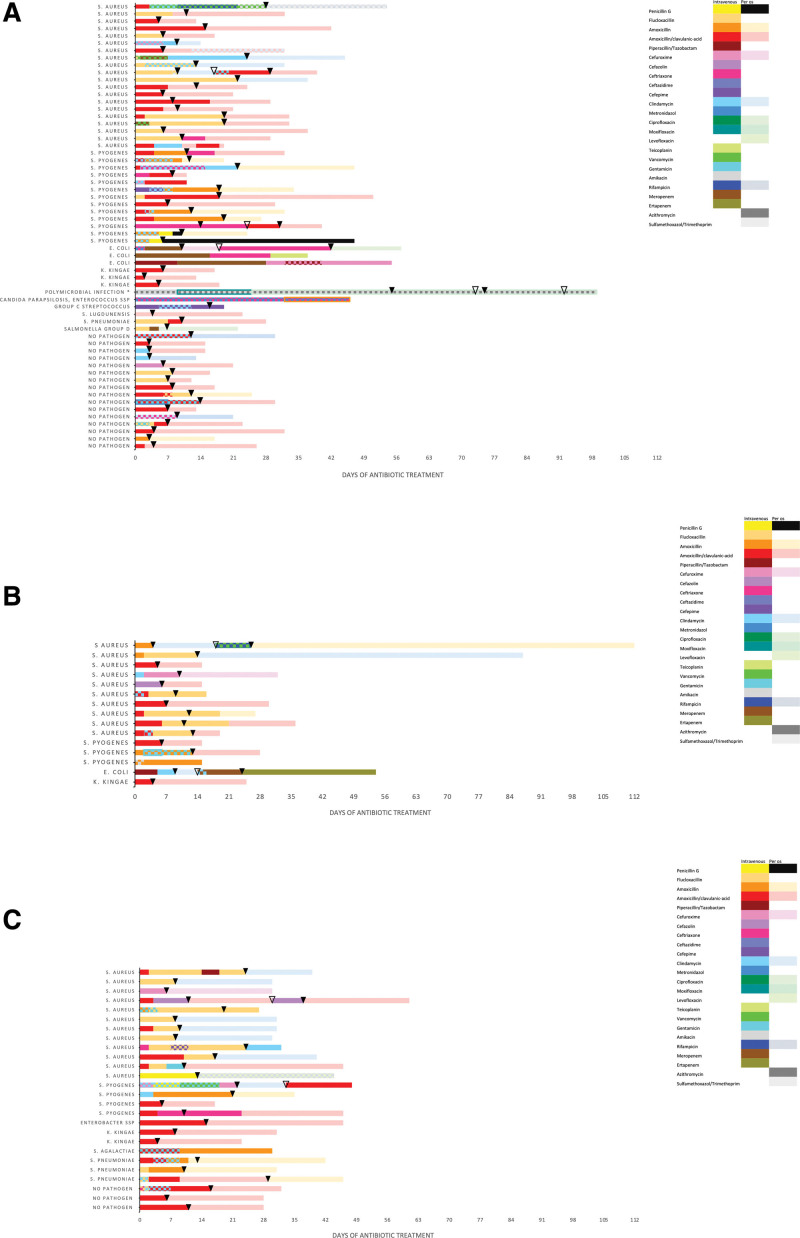
Individual antibiotic treatment (drugs, route and duration) by cultured organisms. A: Pyomyositis. B: Bacterial myositis. C: Associated pyomyositis. Plain colored bars indicate 1 antibiotic substance, square-patterned bars indicate 2 simultaneous antibiotic substances, bordered bars indicate 3 simultaneous antibiotics. ▼: Hospitalization. ▽: Rehospitalization. *Polymicrobial. During the second hospitalization metronidazole, cotrimoxazole, clindamycin were added to the treatment.

All patients were started on empiric antibiotic treatment on admission (Fig. 3A–C). Fifty-one patients (50%) received amoxicillin/clavulanic acid as monotherapy or in combination with clindamycin. Flucloxacillin was used as empiric treatment in 19 patients (19%) and a cephalosporin was given to 14 patients (14%). Overall, median duration of intravenous antibiotic treatment was 30 days (IQR: 19–37) and 15 days (IQR: 9–23) of oral treatment. Median duration of in-hospital antibiotic treatment was 10 days (IQR: 7–17). Antibiotic treatment was continued after hospital discharge in 95 (93%) patients for a median duration of 16 days (IQR: 11–22), generally with oral antibiotics except in 13 patients who continued to receive intravenous antibiotic therapy as out-patients, most often with ceftriaxone (n = 4) or flucloxacillin (n = 4). The median duration of total antibiotic treatment was longer in patients with associated pyomyositis (31.5 days; IQR: 30–44) compared to those with pyomyositis (27 days; IQR: 18–34) and bacterial myositis (27 days; IQR: 16–34).

### Surgical Intervention

In 59 (58%) patients, surgical treatment was performed, including image-guided percutaneous abscess puncture (n = 30) and open surgical abscess drainage (n = 21) (Table 1). Seventeen patients received an intervention that was not related to the pyomyositis, primarily joint punctures (n = 13) in patients classified as SA associated with pyomyositis.

### Course of Disease

The median length of hospitalization was 9 days (IQR: 7–15) (Table 1). Seventeen (17%) patients were admitted to the intensive care unit during hospitalization. Complications occurred in 12 (12%) patients;11 (11%) patients were rehospitalized due to complications due to pyomyositis (n = 6), bacterial myositis (n = 3) and associated pyomyositis (n = 2), respectively, for a median length of 12 days (IQR: 8–13.5). The short-term outcome was favorable with no fatalities and no debilitating sequelae on discharge.

### Special Patient Populations

Of the 13 patients (median age 13.5 years) with an underlying disease, 6 had a malignancy, that is leukemia (n = 3), lymphoma (n = 2) and Ewing sarcoma (n = 1). Five of them were receiving chemotherapy when pyomyositis (n = 4) and bacterial myositis (n = 1) were diagnosed and one patient with OM-associated pyomyositis was newly diagnosed with leukemia at the same time. These patients had a more severe clinical course with 4 of them having multifocal abscesses in up to 10 different muscles. The pathogens were *Escherichia coli* in 3, *S. aureus* in 2 and Group C β-hemolytic streptococcus in one patient.

Five patients initially presented with acute abdomen and were radiologically suspected to have appendicitis; 4 of them underwent laparoscopic appendectomy which rejected the diagnosis. Due to persistent symptoms, an MRI was performed leading to the diagnosis of pyomyositis (4 abscesses in the iliopsoas muscle, 1 abscess of the internal obturator muscle). Two of these patients had an underlying Crohn’s disease and abscess formation in the iliopsoas muscle with possible fistulation. Both were rehospitalized due to recurrent abscesses.

## DISCUSSION

This study is, to our knowledge, the largest series of patients with bacterial myositis or pyomyositis in a nontropical country, comprising 102 patients in the pediatric age group. We found that this disease affects mainly healthy children of all ages, most commonly muscles of the lower extremities and the pelvis, with *S. aureus* and *S. pyogenes* as the main pathogens cultured, and that all patients received antibiotic treatment, although of various regimens and duration.

Similar to other studies,^3,14–16^ we observed an increase in pyomyositis cases over the time period but this was not linked to the emergence of resistant bacterial infections such as community-acquired methicillin-resistant *S. aureus* infections, described by Pannaraj et al^9^ in the United States. It is possible that the increase is due to improved diagnostic procedures, specifically the broader use of MRI, and greater awareness among healthcare professionals. In addition, changes in environmental and lifestyle factors as well as global warming could also be contributing to the fact that the disease, which was once considered a tropical disease, is increasingly prevalent in temperate countries.^3,6,7,14,19^ We did not observe seasonal differences as described for rainy seasons in tropical countries,^6,21,26,27^ which is attributed to the facilitated bacterial skin colonization in humid environments.^7,14^

Pyomyositis is most common in the first two decades of life.^1^ Although most series report male predominance with percentages between 60% and 80%,^3,4,8,12^ we did not observe this in our cohort. About 31% of patients had reported trauma in the previous 4 weeks, mainly affecting the same limb, and 56% had a viral infection. This is in accordance with other reports that identified trauma and other muscle injuries such as rigorous exercise, intramuscular injections and underlying viral myositis^1,5,6,8,21,28^ as predisposing factors. Skeletal muscle, once damaged, becomes susceptible to hematogenous bacterial invasion.^11^ Our patient population consisted of mainly healthy children. Among the 13% with a chronic underlying disease, those 6 with a malignant disease differed most impressively from others, as they were more severely ill and often presented with disseminated disease.

Like other reports,^4–6^ unspecific signs and symptoms such as fever, pain and impaired movement were documented in all our patients. Specific signs, such as swelling of the affected area, were lacking in 62% of the patients, especially in those with pyomyositis affecting deep muscles (6 of 58 patients ie, 10% with pelvic pyomyositis had swelling). Pyomyositis can present in 3 stages: (1) prodromal primary inflammation of the muscle, followed by (2) abscess formation, characterizing “pyomyositis,” potentially with progression to (3) systemic manifestations and sepsis, if left untreated.^6,21^ Most patients (74%) included in this study had stage 2 disease (12% stage 1, 14% stage 3).

In only 20% of our patients was pyomyositis the suspected diagnosis on admission. Rather SA and/or OM were assumed and pyomyositis was usually found by means of MRI and other imaging studies. Of note, 25% still had pyomyositis in association with SA or OM.

In 5 of our patients, appendicitis was suspected and 4 of them had an appendectomy without confirming the diagnosis. Persistence of inflammatory signs prompted further differential diagnostic considerations and tests leading to a diagnosis of pyomyositis. Clinicians should therefore keep in mind that pyomyositis located in the abdomen and pelvis can mimic other, more common diagnoses such as appendicitis or SA of the hip.^6,7,15,19,20,26,28^

As diagnosis of pyomyositis based on clinical grounds is challenging, imaging has an important role in establishing the diagnosis and its distinction from differential diagnoses such as bacterial myositis.^4,18^ We found a clear superiority of MRI in the diagnosis of pyomyositis in this study with the advantage of detecting early inflammatory changes and more detailed determination of the extent and location of the disease.^11^ However, ultrasound and radiograph are more easily accessible and therefore can be used as a primary screening for example SA or trauma.^4^ Also, superficial intramuscular abscess collections can be detected by ultrasound.^11,28^

Initial blood investigations usually reveal signs of inflammation, but they are nonspecific.^6,14,15,28^ Patients with pyomyositis associated with SA or OM showed higher levels of inflammatory markers than patients with pyomyositis or bacterial myositis. In contrast, blood cultures often yield positive results and are helpful in determining targeted antibiotic treatment. We found a higher positivity rate of blood cultures in older children and adolescents. As the time from onset of symptoms to presentation was comparable across all age groups, this difference could possibly be explained by smaller blood volumes taken from children ≤4 years of age. Moreover, *K. kingae* infections are more difficult to detect by blood cultures and are more common in young children. There is one published case of *K. kingae* causing pyomyositis.^29^ Our study adds 3 more such cases plus 1 bacterial myositis and 2 cases of pyomyositis associated with SA.

Cultures of the abscess or surrounding fluids resulted in a high positivity rate (88%). *S. aureus* is the most common causative organism for pyomyositis globally as it was found to be responsible for 50%–95% of all cases.^1–9,11,12,14–16,18–22,26–28,30,31^ Panton-Valentin-Leukocidin (PVL) producing *S. aureus*^4,6,12^ have been associated with more severe courses of the disease with larger abscesses compared to non-PVL producing organisms.^9^ Production of PVL was not systematically tested in *S. aureus* isolates from our retrospective study, therefore we cannot confirm this observation. Beyond *S. aureus*, multiple other bacteria have been implicated, such as Gram-positive (*S. pyogenes* and *S. pneumoniae*) and Gram-negative bacteria (*E.coli*, *Klebsiella pneumoniae*)*28* with notable differences by age of patients. In our study, *S. pyogenes* was detected in 24% of pathogen-positive cases; in fact, it was the most common pathogen in children ≤4 years of age.

Early diagnosis and prompt antibiotic treatment have been suggested as crucial factors influencing favorable outcome, resulting in complete recovery without complications.^4,6,7^ Assigning the course of infection into stages 1–3 can be helpful to define treatment strategies.^28^ While stage 1 disease can be managed with appropriate antibiotics only,^12^ large abscesses often require combined antimicrobial and surgical treatment. In our study, like many other reports,^4,6,8^ surgical abscess drainage was required in over 50% of patients with pyomyositis. Regarding the choice of empiric antibiotic treatment, recommendations are lacking. However, a regimen comparable to treatment of SA or OM seems reasonable,^32^ including antistaphylococcal and antistreptococcal coverage^8^ with modifications depending on local epidemiology and resistance patterns.^9^ In general, the duration of therapy should depend on clinical improvement^9^ and host factors such as immunosuppression.^15^ Treatment for a total of 3–4 weeks seems adequate, ideally initially via intravenous route and switching to an oral antibiotic as soon as possible.^9,28^ However, antibiotic treatment was continued beyond 4 weeks in 56% of our patients.

Patients with pyomyositis showed a longer duration of hospitalization, required more frequent surgical interventions, and showed more complications than patients with bacterial myositis. Possibly, early diagnosis and therapy before the abscess occurs could prevent complications, but early diagnosis in the absence of a detectable abscess is extremely challenging. Reported case fatality ranges between 0 and 10%,^1,4,21,28^ but in general, most patients have excellent recovery. In our study, no fatalities occurred.

A major strength of our study is the large number of cases in the pediatric age group, combined with a standardized approach to data extraction. There are also limitations. First, due to the retrospective design, only recorded information was available for analysis. Second, although we included a broad ICD-10 diagnosis definition, we might have missed cases because of coding errors. Finally, pathogenicity factors of isolates, for example, PVL production, could not be determined retrospectively. In conclusion, pyomyositis should be considered and investigated in any child or adolescent presenting with fever, acute muscle pain and movement restriction, with or without a history of muscle injury. MRI plays a pivotal role in the timely diagnosis and precise localization of the disease. All efforts should be made to obtain microbiological cultures to identify the causative pathogen. With timely and adequate antibiotic therapy and surgical intervention, if needed, most patients have a favorable clinical course.

## ACKNOWLEDGMENTS

*The authors thank the members of the PIGS study group for their onsite support during data extraction and for their participation in the study. Data are available from the corresponding author by request.*

## Supplementary Material


