# Supplementary material for: Clinical Characteristics and Management of Children and Adolescents Hospitalized With Pyomyositis
Source: Pediatr Infect Dis J. 2024 May 16;43(9):831–40. doi: 10.1097/INF.0000000000004382 (PMC11319086; doi:10.1097/INF.0000000000004382)

**Supplemental Digital Content 1.** Localization of bacterial myositis or pyomyositis per muscle group (37 patients had more than one affected muscle)


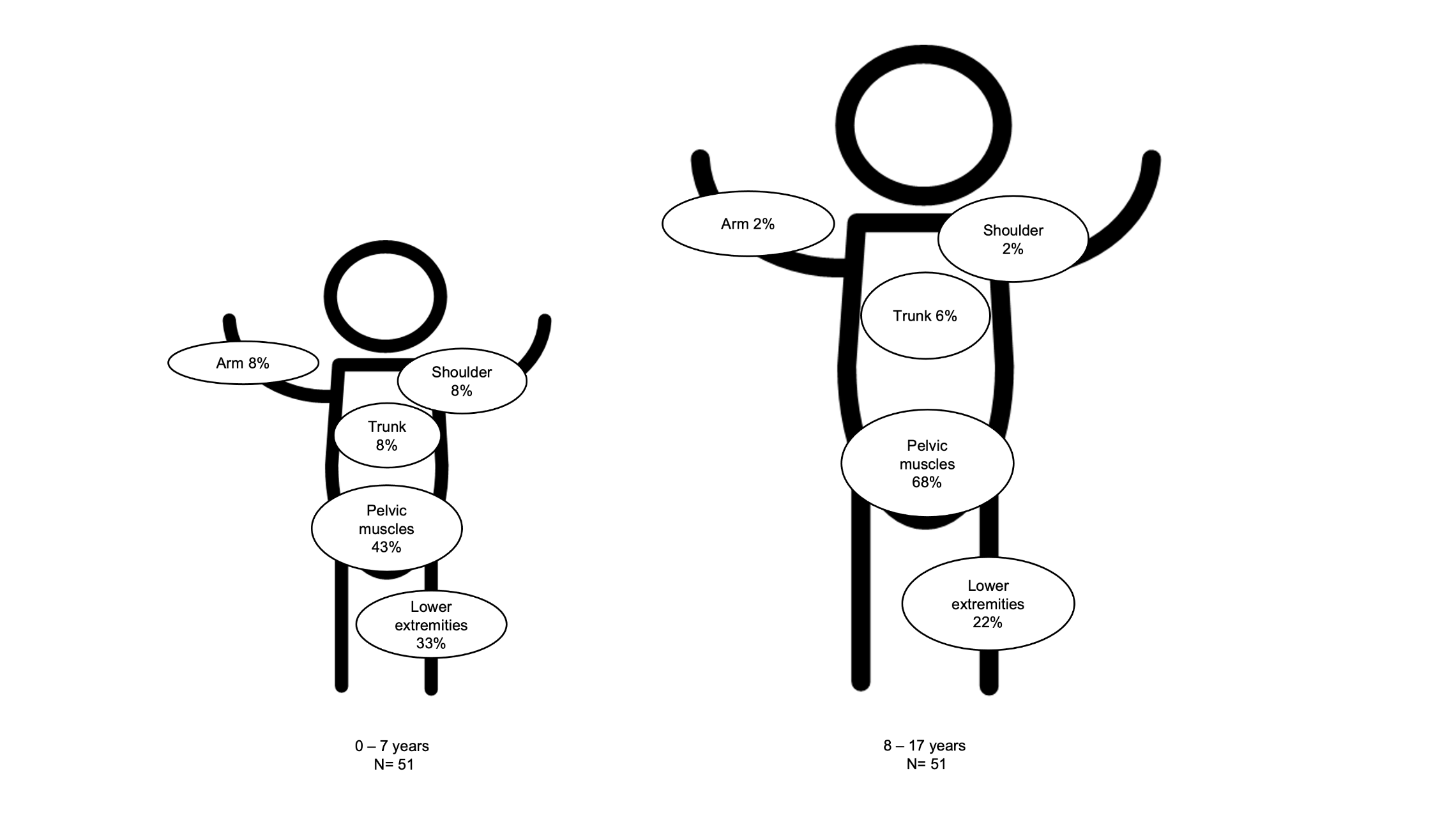

Supplement: Supplementary file 1 [file inf-43-831-s001.docx]
