# Supplementary material for: Clinical Characteristics and Management of Children and Adolescents Hospitalized With Pyomyositis
Source: Pediatr Infect Dis J. 2024 May 16;43(9):831–40. doi: 10.1097/INF.0000000000004382 (PMC11319086; doi:10.1097/INF.0000000000004382)

**Supplemental Digital Content 2.**  White blood cell count (WBC) values at admission by diagnosis classification for pyomyositis, bacterial myositis, or associated pyomyositis.

**
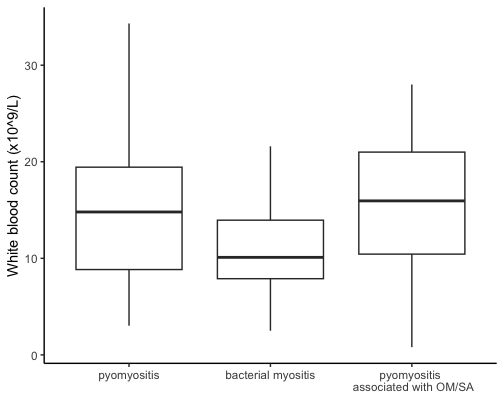
**

**Supplemental Digital Content 3.** C-reactive protein (CRP) values at admission by diagnosis classification for pyomyositis, bacterial myositis, or associated pyomyositis.

**
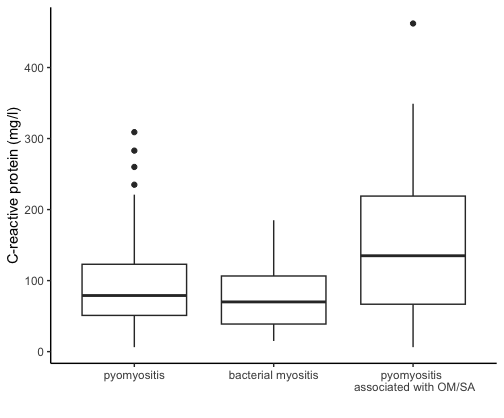
**

**Supplemental Digital Content 4.** Erythrocyte sedimentation rate (ESR) values at admission by diagnosis classification for pyomyositis, bacterial myositis, or associated pyomyositis.


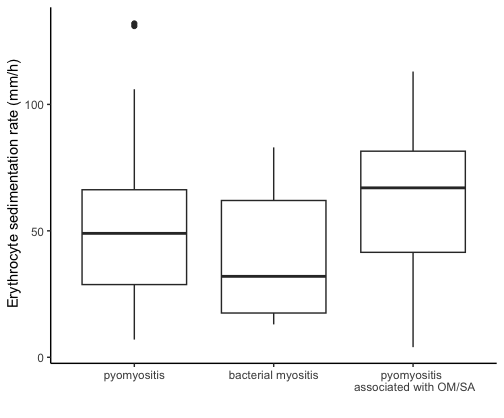

Supplement: Supplementary file 2 [file inf-43-831-s002.docx]
